# Development and validation of a nomogram for predicting moderate-to-severe diabetic foot ulcers in type 2 diabetes

**DOI:** 10.3389/fendo.2025.1669605

**Published:** 2025-10-06

**Authors:** Jinying Zhang, Jing Lin, Lizhen Wu, Jiayu Lin

**Affiliations:** ^1^ Department of Geriatrics, The Second Affiliated Hospital of Fujian Medical University, Quanzhou, Fujian, China; ^2^ The School of Basic Medical Sciences, Fujian Medical University, Fuzhou, Fujian, China; ^3^ Department of Endocrinology, The Second Affiliated Hospital of Fujian Medical University, Quanzhou, Fujian, China

**Keywords:** diabetes mellitus, type 2, nomogram, diabetic foot ulcer, data analysis, logistic models

## Abstract

**Background:**

Diabetic foot ulcer (DFU) has become a significant public health concern. This research aimed to develop a predictive nomogram model to assess the risk of moderate to severe DFU in patients with diabetes.

**Methods:**

Our retrospective study included 499 hospitalized patients with Type 2 Diabetes Mellitus (T_2_DM) and moderate to severe DFUs at the Second Affiliated Hospital of Fujian Medical University, from January 2021 to December 2023. Predictive factors were assessed using both univariate and multivariate logistic regression analyses, leading to the establishment of the predictive nomogram. The model’s performance was evaluated through receiver operating characteristic (ROC) curves, calibration curves, decision curve analysis, and clinical impact curve.

**Results:**

The predictive model included several risk factors: diabetic retinopathy (DR), diabetic kidney disease (DKD), diabetic peripheral neuropathy (DPN), peripheral angiopathy (PAD), D-dimer, K-time, total cholesterol (TC), Low-Density Lipoprotein cholesterol (LDL-C), and high-density lipoprotein cholesterol (HDL-C). The model demonstrated excellent discrimination, with AUC 0.977 (95% CI: 0.965–0.989) in the training cohort and 0.977 (95% CI: 0.958–0.996) in the validation cohort. Calibration results indicated strong agreement between predicted and observed outcomes. Additionally, decision curve analysis indicated that the nomogram provided clinical benefits in both the training and validation cohorts.

**Conclusion:**

This nomogram, which incorporates DKD, DPN, PAD, DR, D-dimer, K-time, TC, LDL-C, and HDL-C, demonstrates strong accuracy and predictive value for assessing the risk of moderate to severe DFUs in patients with diabetes.

## Introduction

Diabetic foot ulcer (DFU) refers to the condition characterized by ulcers and gangrene in the feet of patients with diabetes, primarily due to microcirculatory insufficiency resulting from large vascular and microvascular lesions (1). According to the International Diabetes Federation (IDF), approximately 18.6 million patients with diabetes develop a DFU each year. Throughout their lifetime, about 34% of individuals with type 1 or type 2 diabetes will experience a DFU (2). The Wagner grading system is a crucial tool for assessing the severity of DFUs. It classifies DF conditions into six levels, ranging from 0 to 5. Based on this grading, further subdivisions can be established to differentiate between mild DF (levels 0-2) and moderate to severe DFUs (levels 3-5) (3). This classification aids healthcare providers in determining the appropriate treatment and management strategies for patients with DFUs (4). Up to 20% of individuals with a DFU may need hospitalization, and between 15% and 20% of those hospitalized may undergo a lower extremity amputation (5, 6). The primary causes of lower extremity amputation are infection and progressive gangrene (7), with around 50% of DFUs becoming infected (8). It is important to note that lower extremity amputations are associated with moderate to severe diabetic foot conditions. Furthermore, a study of patients with diabetes revealed a crude mortality rate of 231 deaths per 1,000 person-years among individuals with a DFU, compared to 182 deaths per 1,000 person-years in those without a DFU (9).

Several recent studies have identified risk factors associated with DFUs, including inadequate glycemic control, poor renal function, peripheral neuropathy, and peripheral vascular disease (10). Recognizing these risk factors is essential for prevention and early intervention, as it can help reduce the incidence of amputations, improve patients’ quality of life, and lessen the burden on the healthcare system. However, the early identification of moderate to severe diabetic foot ulcers remains challenging.

K-time, a parameter from thromboelastography (TEG), which quantifies the speed of fibrin buildup and clot strengthening by measuring the time from the start of clot formation until the TEG trace amplitude reaches 20 mm (11). A short K-time, indicating accelerated fibrin polymerization and clot strengthening, coupled with elevated D-dimer, indicating enhanced fibrin breakdown, is characteristic of a hypercoagulable state (11). In the context of diabetes-induced endothelial dysfunction and peripheral arterial disease, this prothrombotic milieu is hypothesized to contribute to microvascular thrombosis in the foot. The occlusion of small vessels further compromises already diminished perfusion, exacerbating tissue ischemia, impairing wound healing, and ultimately facilitating the progression to more severe ulcer stages (11). To our knowledge, no nomogram is currently available that integrates coagulation parameters (such as K-time and D-dimer) with microvascular complications and lipid profiles for predicting DFU severity. Therefore, we aim to develop a predictive nomogram model (12) to assess the risk of moderate to severe DFU in patients with diabetes. This model will serve as a practical tool for early identification of at-risk individuals, thereby aiding clinicians in formulating timely and personalized preventive strategies.

## Materials and methods

### Participants

A total of 499 patients with Type 2 Diabetes Mellitus (T2DM) were consecutively screened and enrolled in our retrospective study. The Wagner classification was used to grade ulcer severity. This assessment was based on the clinical evaluation performed by the treating physician at the time of presentation, as documented in the patient’s medical record. Among them, 248 patients presented with moderate-to-severe DFUs (classified as Wagner grades 3–5), while the remaining 251 patients had T2DM but did not have moderate-to-severe DFUs. These patients were hospitalized at the Second Affiliated Hospital of Fujian Medical University between January 2021 and December 2023. This study was reviewed and authorized by the Ethics Committee at the Second Hospital of Fujian Medical University, and it adhered to the principles of the Declaration of Helsinki.

Inclusion criteria were as follows: (1) Patients who meet the diagnostic criteria for T_2_DM (13), (2) Patients with DFUs who meet the diagnostic criteria for Wagner grades ranging from 3 to 5 (3), (3) Patients over 18 years of age, (4) Complete and reliable clinical medical records. Exclusion criteria were as follows: (1) Pregnant or lactating women. (2) Patients with significant dysfunction in multiple organ systems, this included: ① end-stage renal disease requiring dialysis; ② severe heart failure (New York Heart Association Class III or IV); ③ severe liver cirrhosis (Child-Pugh class C); ④ advanced respiratory failure requiring home oxygen therapy.

### Data collection

Clinical data was extracted from the electronic medical records within the hospital information system, encompassing general demographic information such as gender, age, body mass index (BMI), diastolic and systolic blood pressure (DBP and SBP), and family history. The data also included comorbidities like hypertension, coronary heart disease (CHD), stroke, and a history of diabetic complications, which comprised diabetic retinopathy (DR), diabetic kidney disease (DKD), diabetic peripheral neuropathy (DPN), and peripheral angiopathy (PAD). The medication history includes lipid-lowering drugs, antiplatelet drugs, oral hypoglycemic drugs, insulin, and the use of insulin pumps. Blood examination results upon admission included C-reactive protein (CRP), hemoglobin A1c (HbA1c), fasting blood glucose (FBG), white blood cells (WBC), monocytes, lymphocytes, platelets (PLT), total cholesterol (TC), triglycerides (TG), Low-Density Lipoprotein cholesterol (LDL-C), high-density lipoprotein cholesterol (HDL-C), serum creatinine (SCr), blood uric acid (BUA), D-dimer, fibrinogen (Fib), Angle-α, and K-time. To ensure data quality, all information was input and cross-checked in parallel by two individuals.

### Modeling process

Patients were randomly assigned to a training cohort and a validation cohort in a ratio of 3:1. Primary predictive factors were evaluated using both univariate and multivariate logistic regression analyses to determine their impact as independent predictors for DFUs in patients with diabetes. Based on these independent influential factors, a predictive model was constructed. The performance of the Nomogram forecast model was assessed using the Receiver Operating Characteristic (ROC) curve. The accuracy of the model was validated using the calibration curve. The clinical utility of the nomogram was evaluated through Decision Curve Analysis (DCA) and Clinical Impact Curve (CIC), which analyzed the net benefit at various risk thresholds.

### Statistical analysis

All statistical analyses were performed using SPSS version 26.0 and R version 4.2.2. Continuous variables that followed a normal distribution were expressed as mean ± standard deviation (mean ± SD), while categorical variables were presented as n (%). Continuous variables were analyzed using Student’ s t-test or one-way ANOVA, as appropriate. The T-test was employed for continuous variables with a normal distribution, and the non-parametric rank sum test (Wilcoxon) was used for continuous variables that did not comply with a normal distribution. The chi-squared test was utilized for comparisons between groups. The “createDataPartition” function from the “caret” package was utilized to split the dataset into training and testing sets in a 3:1 ratio. The ROC curve for predicting binary classification variables was implemented using the “pROC” package in R. Figures were generated using the “ggplot2”, “rmda”, and “Regplot” packages. Multicollinearity among the predictor variables was assessed using the variance inflation factor (VIF), with a value of 10 set as the threshold for indicating severe multicollinearity. Internal validation of the model was conducted using bootstrapping techniques to correct for optimism bias. All statistical data were analyzed using a two-sided test, with a p-value < 0.05 considered statistically significant.

## Results

### Clinical characteristics

A total of 499 patients were enrolled in this study, comprising 374 patients in the training cohort and 125 patients in the validation cohort (**Table 1**). Among the participants, there were 312 males (62.53%) and 187 females (37.47%). The average age of the patients was 59.66 ± 13.56 years, and the average body mass index (BMI) was 23.63 ± 4.00 kg/m². **Table 1** presented an overview of the relevant clinical information for the two cohorts. No statistically significant differences were observed between the cohorts for other clinical factors, except for diastolic blood pressure (DBP) and fasting blood glucose (FBG) (p = 0.0349 and 0.0478, respectively) (**Table 1**).

**Table 1:** General clinical characteristics.

Variable	Level	All patients (n = 499)	Validation cohort (n=125)	Training cohort (n=374)	*P* value
n		499	125	374	
Gender (%)	Female	187 (37.47)	55 (44.00)	132 (35.29)	0.102
	Male	312 (62.53)	70 (56.00)	242 (64.71)	
Age (years)		59.66 (13.56)	60.14 (13.59)	59.51 (13.57)	0.65
BMI (kg/m^2^)		23.63 (4.00)	23.65 (4.15)	23.62 (3.95)	0.93
SBP (mmHg)		132.10 (20.21)	132.03 (19.66)	132.12 (20.42)	0.97
DBP (mmHg)		78.84 (11.25)	76.98 (10.51)	79.45(11.44)	0.03
Family history (%)	No	402 (80.56)	99 (79.20)	303 (81.02)	0.75
	Yes	97 (19.44)	26 (20.80)	71 (18.98)	
HBP (%)	No	257 (51.50)	59 (47.20)	198 (52.94)	0.31
	Yes	242 (48.50)	66 (52.80)	176 (47.06)	
CAD (%)	No	392 (78.56)	96 (76.80)	296 (79.14)	0.67
	Yes	107 (21.44)	29 (23.20)	78 (20.86)	
Stroke (%)	No	451 (90.38)	109 (87.20)	342 (91.44)	0.22
	Yes	48 (9.62)	16 (12.80)	32 (8.56)	
DKD (%)	No	335 (67.13)	79 (63.20)	256 (68.45)	0.33
	Yes	164 (32.87)	46 (36.80)	118 (31.55)	
DPN (%)	No	86 (17.23)	25 (20.00)	61 (16.31)	0.42
	Yes	413 (82.77)	100 (80.00)	313 (83.69)	
DR (%)	No	258 (51.70)	70 (56.00)	188 (50.27)	0.32
	Yes	241 (48.30)	55 (44.00)	186 (49.73)	
PAD (%)	No	273 (54.71)	74 (59.20)	199 (53.21)	0.29
	Yes	226 (45.29)	51 (40.80)	175 (46.79)	
Amputation history (%)	No	443 (88.78)	110 (88.00)	333 (89.04)	0.88
	Yes	56 (11.22)	15 (12.00)	41 (10.96)	
Use of lipid lowering drugs (%)	No	140 (28.06)	35 (28.00)	105 (28.07)	1.00
	Yes	359 (71.94)	90 (72.00)	269 (71.93)	
Use of antiplatelet drugs (%)	No	338 (67.74)	80 (64.00)	258 (68.98)	0.36
	Yes	161 (32.26)	45 (36.00)	116 (31.02)	
Use of hypoglycemic drugs (%)	No	174 (34.87)	42 (33.60)	132 (35.29)	0.81
	Yes	325 (65.13)	83 (66.40)	242 (64.71)	
Use of insulin (%)	No	73 (14.63)	18 (14.40)	55 (14.71)	1.00
	Yes	426 (85.37)	107 (85.60)	319 (85.29)	
Use of insulin pump (%)	No	392 (78.56)	102 (81.60)	290 (77.54)	0.41
	Yes	107 (21.44)	23 (18.40)	84 (22.46)	
FBG (mmol/L)		8.12 (3.65)	7.56 (3.15)	8.31 (3.79)	0.05
HbA1c (%)		8.52 (2.34)	8.38 (2.02)	8.56 (2.44)	0.46
WBC (10^^9^/L)		9.24 (5.08)	9.19 (4.76)	9.26(5.18)	0.90
Mono (10^^9^/L)		6.60 (2.08)	6.49 (1.72)	6.64 (2.19)	0.47
Lymph (10^^9^/L)		23.78 (11.45)	23.92(11.22)	23.74(11.54)	0.88
PLT (10^^9^/l)		269.97 (103.09)	272.93(104.76)	268.98(102.65)	0.71
Fib (g/l)		4.54 (1.96)	4.25 (1.90)	4.64 (1.97)	0.05
D-dimer (ng/ml)		1.01 (1.83)	1.11 (2.33)	0.98 (1.63)	0.48
Angle-α (°)		67.50 (9.26)	67.11(9.83)	67.63(9.07)	0.59
K-time (min)		1.50 (0.61)	1.53 (0.63)	1.50 (0.60)	0.65
ALT (U/L)		24.19 (45.81)	27.58(72.00)	23.06(32.77)	0.34
AST (U/L)		24.15 (67.36)	24.75(53.58)	23.95(71.44)	0.91
TC (mmol/L)		4.39 (1.54)	4.44 (1.97)	4.35 (1.37)	0.57
TG (mmol/L)		1.79 (1.69)	1.85 (1.99)	1.77(1.58)	0.65
LDL-C (mmol/L)		2.63 (1.20)	2.64(1.33)	2.63(1.15)	0.94
HDL-C (mmol/L)		1.02 (0.37)	1.03 (0.42)	1.02 (0.35)	0.77
SCr (umol/L)		159.34(178.18)	161.95 (164.82)	158.47 (182.62)	0.85
BUA (umol/L)		323.32 (117.65)	313.84 (115.71)	326.48 (118.27)	0.30
UACR (mg/g)		133.92 (211.15)	120.12 (182.42)	138.54 (219.95)	0.40

n, number of patients; BMI, body mass index; SBP, Systolic Blood Pressure; DBP, Diastolic Blood Pressure; HBP, Hypertension; CAD, Coronary Artery Disease; DKD, Diabetic Kidney Disease; DPN, diabetic peripheral neuropathy; DR, diabetic retinopathy; PAD, peripheral angiopathy of diabetic; FBG, fasting blood glucose; HbA1c, Hemoglobin A1c; WBC, white blood cells; Mono, Monocyte; Lymph, Lymphocyte; PLT, platelet; Fib, fibrinogen; K-time, Kinetics time; Alanine Aminotransferase; AST, Aspartate Aminotransferase; TC, Total cholesterol; TG, Triglycerides; LDL-C, Low-Density Lipoprotein cholesterol; HDL-C, High-Density Lipoprotein cholesterol; SCr, Serum creatinine; BUA, blood uric acid; UACR, Urinary Albumin to Creatinine Ratio.

### Univariate and multivariate logistic regression analysis

Univariate logistic regression analysis revealed several risk factors with statistically significant differences, including age, BMI, SBP, DBP, Family history, HBP, CAD, stroke, DKD, DPN, DR, PAD, FBG, HbA1c, WBC, monocytes, lymphocytes, PLT, Fib, D-dimer, Angle-α, K-time, TC, TG, LDL-C, HDL-C, UACR, use of antiplatelet drugs, and use of hypoglycemic drugs (P <0.05, **Table 2**).

**Table 2:** Univariate and multivariate logistic regression analysis.

Variable	β Value	OR (95% CI)	P Value
Univariate analysis			
Gender	0.15	1.16 (0.76-1.77)	0.500
Age	0.07	1.08 (1.06 - 1.10)	**<0.001**
BMI	0.23	1.26 (1.18-1.35)	**<0.001**
SBP	0.02	1.02 (1.01 - 1.03)	**0.002**
DBP	0.02	1.02 (1.01-1.04)	**0.010**
Family history	1.58	4.83 (2.65-8.81)	**<0.001**
HBP	0.66	1.93 (1.28-2.92)	**0.002**
CAD	1.99	7.30 (3.80-14.03)	**<0.001**
Stroke	2.02	7.52 (2.58-21.74)	**<0.001**
DKD	0.98	2.67 (1.68 - 4.22)	**<0.001**
DPN	0.40	30.30 (9.17 - 100.00)	**<0.001**
DR	1.89	6.58 (4.20 - 10.31)	**<0.001**
PAD	3.08	21.74 (12.66 - 37.04)	**<0.001**
FBG	0.14	1.15 (1.08 - 1.23)	**<0.001**
HbA1c	0.17	1.19 (1.09 - 1.30)	**<0.001**
WBC	0.33	1.39 (1.27 - 1.52)	**<0.001**
Mono	0.04	1.04 (0.94-1.14)	0.459
Lymph	0.15	1.16 (1.13-1.20)	**<0.001**
PLT	0.01	1.01 (1.00 - 1.01)	**<0.001**
Fib	0.91	2.48 (2.02 - 3.03)	**<0.001**
D-dimer	3.27	26.32 (11.91 - 58.82)	**<0.001**
Angle-α	0.26	1.30 (1.24 - 1.37)	**<0.001**
K-time	-4.87	0.01 (0.003 - 0.02)	**<0.001**
ALT	0.003	1.00 (0.996-1.01)	0.440
AST	-0.001	1.00 (0.995-1.00)	0.546
TC	-0.39	0.68 (0.58 - 0.80)	**<0.001**
TG	0.35	1.42 (1.17-1.73)	**<0.001**
LDL	0.35	1.41 (1.17-1.71)	**<0.001**
HDL	1.12	3.06 (1.63-5.74)	**<0.001**
Cr	0.001	1.00 (0.999-1.00)	0.398
UA	-0.001	0.999 (0.998-1.00)	0.464
UACR	-0.002	0.998 (0.997-0.999)	**<0.001**
Use of lipid-lowering drugs	-0.38	0.68 (0.43-1.07)	0.099
Use of antiplatelet drugs	-1.16	0.31 (0.20-0.50)	**<0.001**
Use of hypoglycemic drugs	1.17	3.21 (2.05-5.04)	**<0.001**
Use of insulin	-0.29	0.75 (0.42-1.33)	0.324
Use of insulin pump	0.14	1.16 (0.71-1.88)	0.561
Multivariate analysis			
DKD	1.26	3.54 (1.36-10.21)	**0.018**
DPN	1.87	6.45 (1.42-37.04)	**0.012**
DR	0.89	2.44 (1.98-6.21)	**0.017**
PAD	2.88	17.86 (6.90-52.63)	**<0.001**
D-dimer	1.52	4.57(1.96-13.33)	**0.007**
K-time	-1.16	0.31 (0.08-0.84)	**0.009**
TC	-3.96	0.02 (0.005-0.06)	**0.012**
LDL-C	1.20	3.31 (1.21-8.89)	**0.020**
HDL-C	0.96	2.60 (1.83-8.00)	**0.016**

BMI, body mass index; SBP, Systolic Blood Pressure; DBP, Diastolic Blood Pressure; HBP, Hypertension; CAD, Coronary Artery Disease; DKD, Diabetic Kidney Disease; DPN, diabetic peripheral neuropathy; DR, diabetic retinopathy; PAD, peripheral angiopathy of diabetic; FBG, fasting blood glucose; HbA1c, Hemoglobin A1c; WBC, white blood cells; Mono, Monocyte; Lymph, Lymphocyte; PLT, platelet; Fib, fibrinogen; K-time, Kinetics time; Alanine Aminotransferase; AST, Aspartate Aminotransferase; TC, Total cholesterol; TG, Triglycerides; LDL-C, Low-Density Lipoprotein cholesterol; HDL-C, High-Density Lipoprotein cholesterol; SCr, Serum creatinine; BUA, blood uric acid; UACR, Urinary Albumin to Creatinine Ratio.

The bold values indicate statistical significance, representing a *p*-value of < 0.05.

To avoid omitting important variables, identify hidden interactions, and control for confounding factors, these risk factors were subsequently incorporated into the multivariate logistic regression analysis. The result indicated that the independent risk factors for moderate to severe DFUs among patients were DKD, DPN, DR, PAD, D-dimer, K-time, TC, LDL-C, and HDL-C (P < 0.05, **Table 2**). All variables had VIF values below 10, and the tolerance was above 0.1, indicating no significant multicollinearity among these factors.

### Development of nomogram prediction model

**Figure 1:**
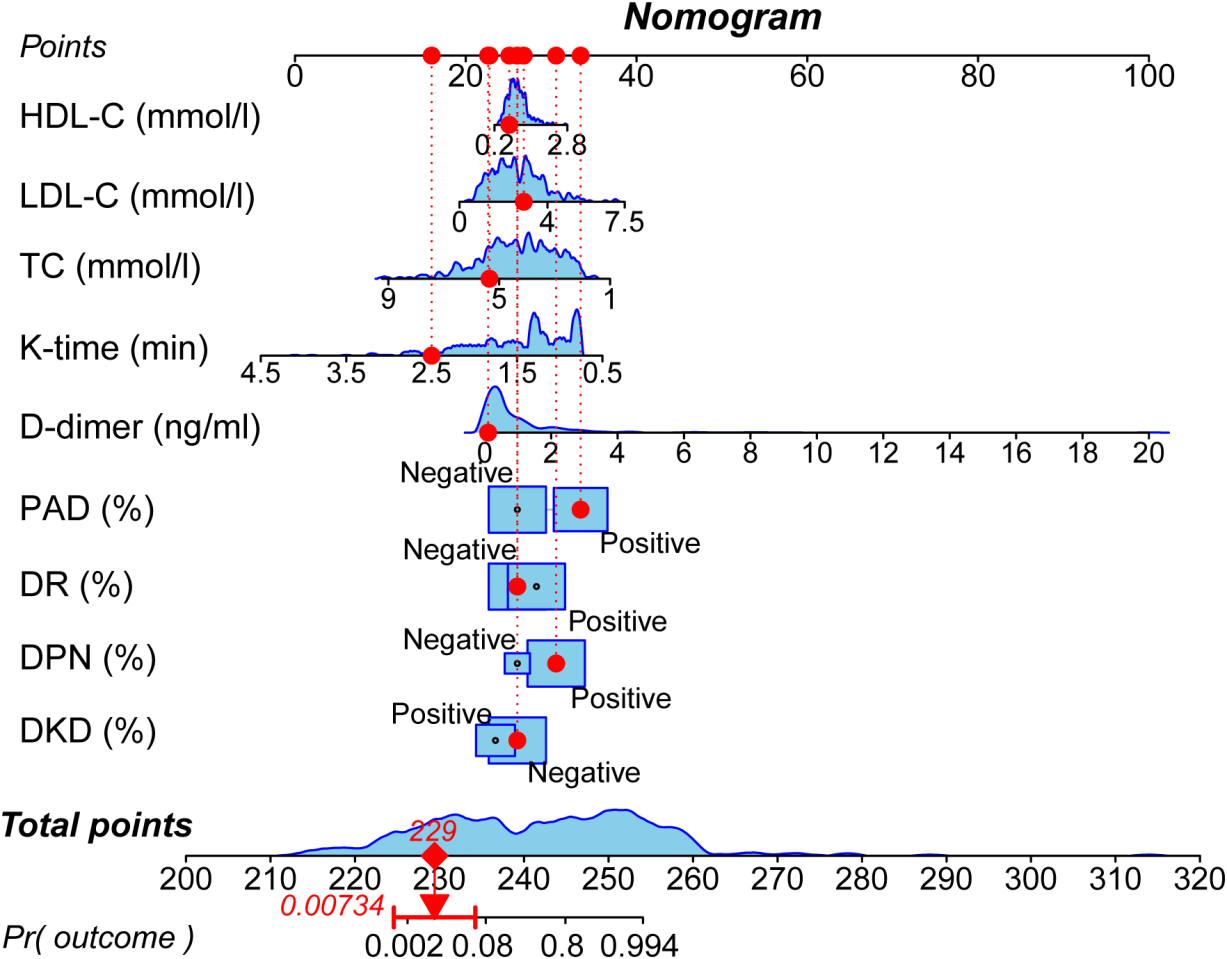
Nomogram for predicting the risk of moderate to severe DFUs. The nomogram incorporates nine independent predictors identified through multivariate analysis: DKD, DPN, DR, PAD, D-dimer level, K-time, TC, LDL-C, and HDL-C. To use the nomogram, locate the patient’s value for each predictor, draw a line upward to the ‘Points’ axis to determine the corresponding score, sum all the points, and then locate the total on the ‘Total Points’ axis. A line drawn downward to the ‘Risk’ axis will indicate the individual’s predicted probability of having a moderate to severe DFU.

Based on the results of the multivariate logistic regression analysis, a nomogram model was established to predict the risk of moderate to severe DFUs in patients with T_2_DM using the identified independent risk factors (**Figure 1**). The horizontal axis represented total points that can be accumulated from the individual risk factors. Points were calculated by plotting the values of these factors on their respective scales and summing them. The nomogram included a probability scale at the bottom, indicating the likelihood of developing DFUs, ranging from very low probabilities (0.002) to near certainty (0.994). For instance, if a patient had DKD contributing 30 points, DPN adding 25 points, and DR contributing 20 points, the total would be 75 points. This total was then used to find the corresponding probability of developing moderate to severe DFUs, typically ranging from 0% to 100%.

### Validation of the nomogram prediction model

**Figure 2:**
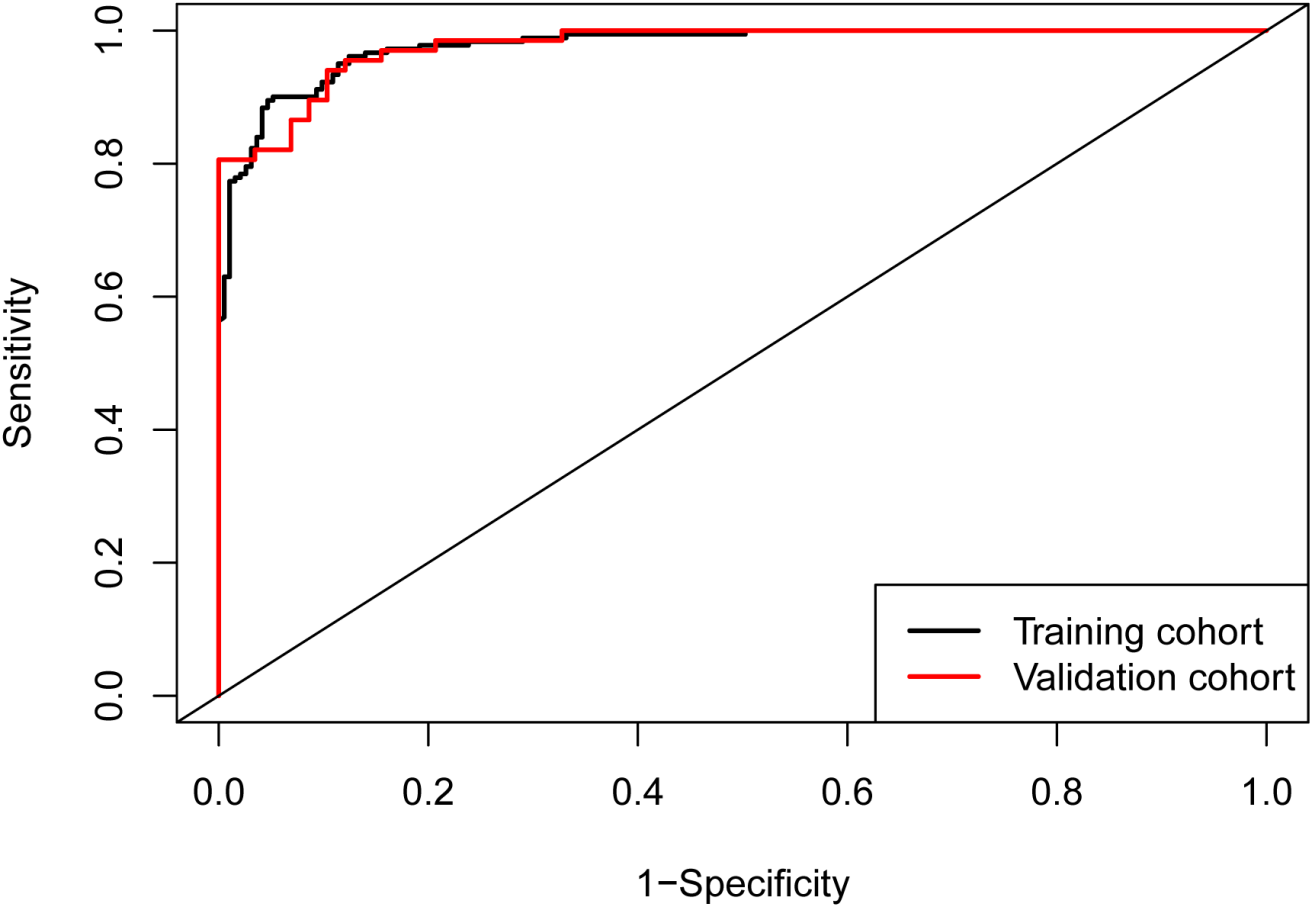
ROC curves of the nomogram. The area under the curve (AUC) was 0.977 (95% CI: 0.965–0.989) in the training cohort and 0.977 (95% CI: 0.958–0.996) in the validation cohort, demonstrating excellent discriminatory ability of the model in both cohorts.

ROC curves were generated for both the training cohort and the validation cohort (**Figure 2**). The training cohort demonstrated an area under the curve (AUC) of 0.977 (95% CI: 0.965–0.989). The Youden index was approximately 0.849, with a cutoff value of 0.538, yielding a sensitivity of 94.8% and specificity of 90.1%. Similarly, in the validation cohort, the AUC was also 0.977 (95% CI: 0.958–0.996). The Youden index was approximately 0.837, with a cutoff value of 0.483, yielding a sensitivity of 89.7% and specificity of 94.0%. Both cohorts exhibited AUC values greater than 0.75, indicating strong model discrimination.

**Figure 3:**
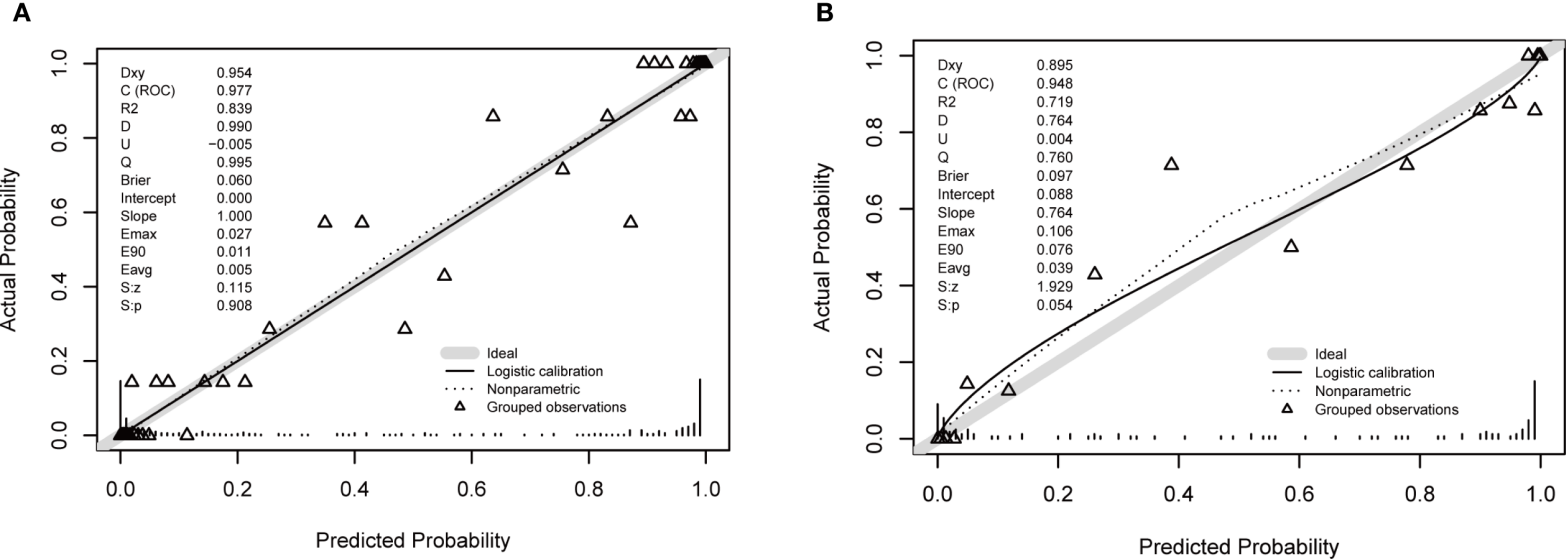
Calibration plots for the nomogram. **(A)** Training cohort. **(B)** Validation cohort. The plots compare the predicted probability of moderate to severe DFU (x-axis) against the observed frequency (y-axis). The solid line represents the performance of the nomogram, and the dashed line represents the ideal reference line where predictions perfectly match observations. The close alignment of the solid line to the dashed line, along with a non-significant Hosmer-Lemeshow test result (p > 0.05), indicates good calibration of the model.

Calibration curves for both cohorts illustrated good consistency between predicted and actual probabilities (**Figures 3A, B**). The Brier scores were 0.06 for the training cohort and 0.097 for the validation cohort. These Brier scores, being closest to 0, indicated the excellent performance of the constructed prediction model.

### Clinical application

**Figure 4:**
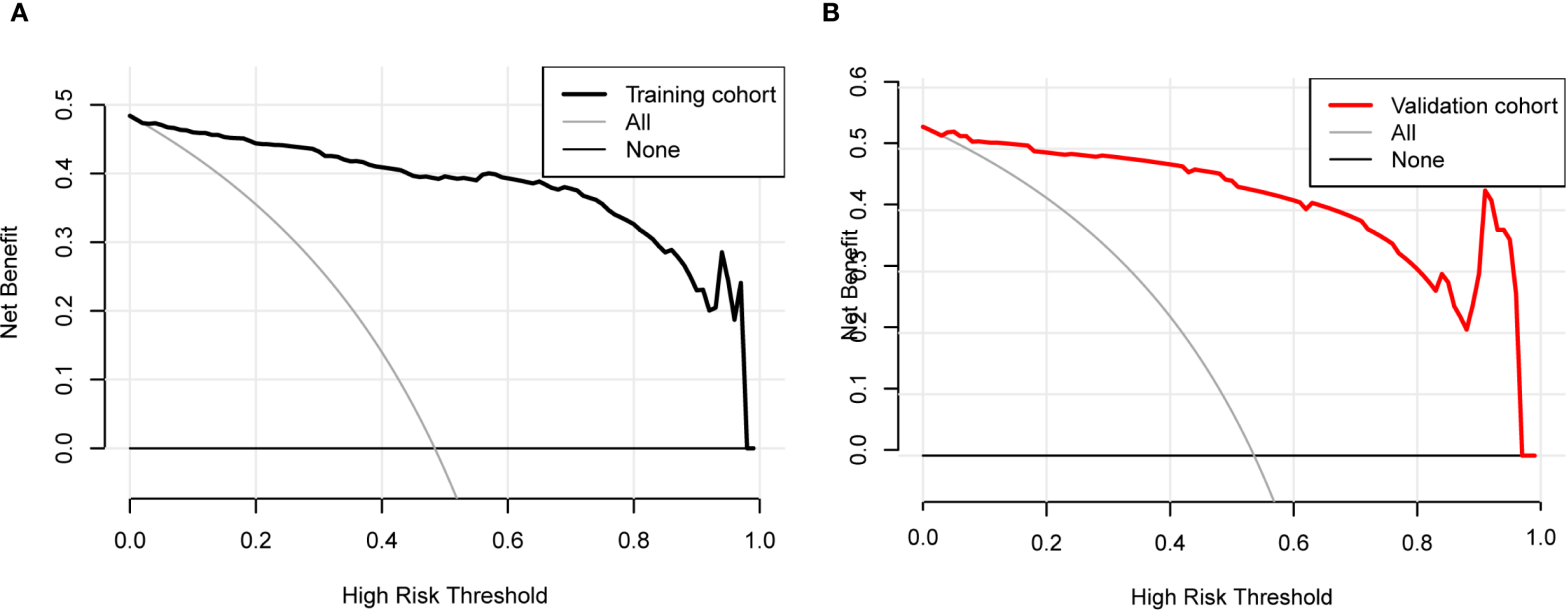
Decision curve analysis (DCA) for the nomogram. **(A)** Training cohort. **(B)** Validation cohort. The DCA evaluates the clinical utility of the nomogram by plotting the net benefit (y-axis) against the threshold probability (x-axis). The strategy of using the nomogram for intervention decisions provides a greater net benefit than the ‘treat all’ or ‘treat none’ strategies across a wide range of clinically reasonable threshold probabilities.

**Figure 5:**
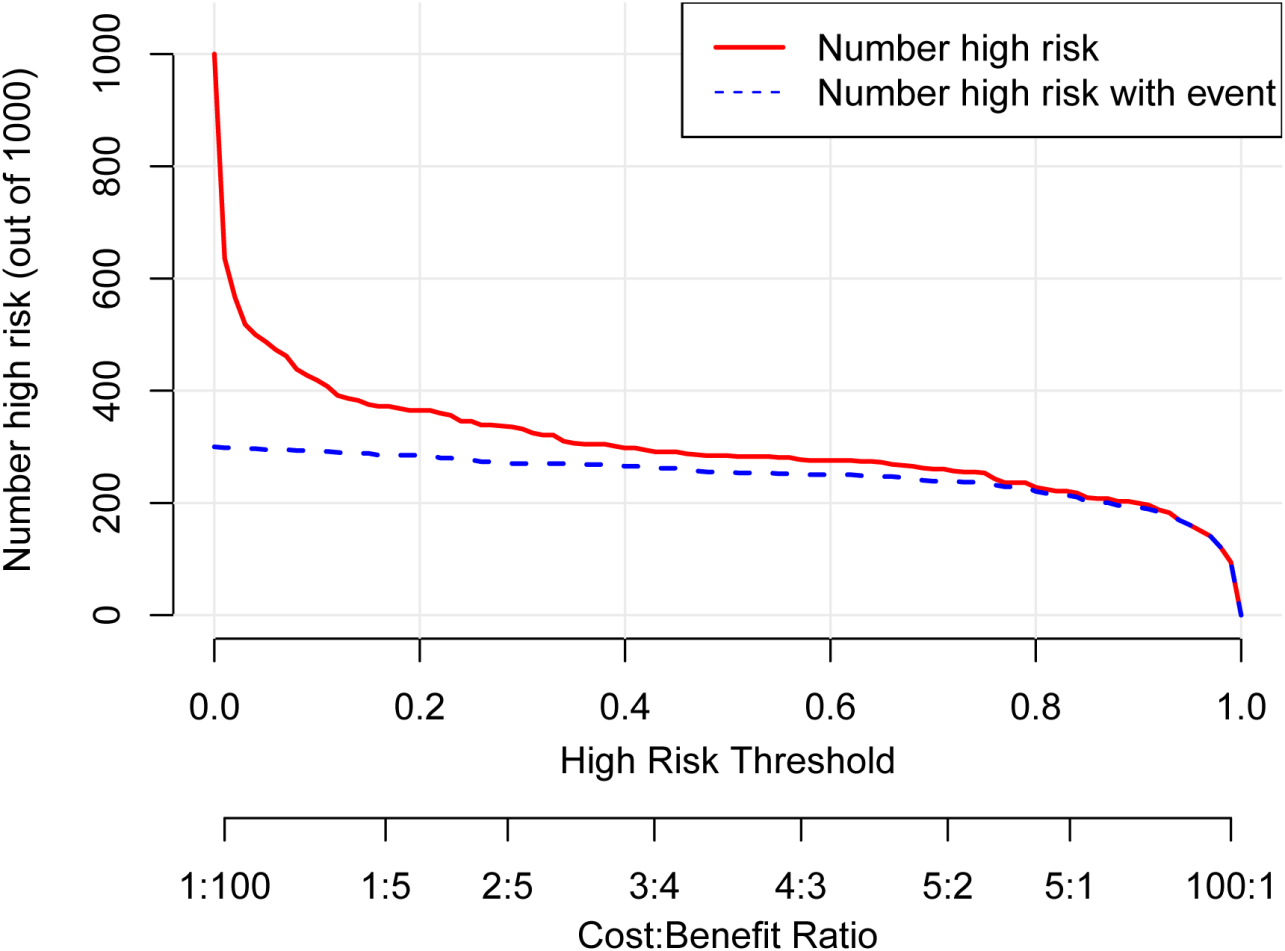
Clinical impact curve (CIC) for the nomogram. The CIC evaluates the clinical utility of the nomogram by plotting the number of people classified as high risk (red line) against the number of true positives (blue line) at different risk thresholds. The close correspondence between the two lines across most thresholds indicates that the model effectively identifies true positive cases without overestimating the high-risk population, supporting its practical value for risk stratification.

Decision Curve Analysis (DCA) curves were generated for both the training cohort and the validation cohort (**Figures 4A, B**). In the threshold probability ranges of 0%–100%, the net benefit levels associated with the nomogram prediction model significantly exceeded those of other options, demonstrating superior clinical utility. Within these ranges, the nomogram prediction model shows strong clinical applicability. The Clinical Impact Curve (CIC) was plotted to assess the practical clinical usefulness of the nomogram prediction model (**Figure 5**). When the threshold probability exceeds 0.4, the model’s predictions demonstrate a high level of agreement with actual clinical outcomes, indicating a strong predictive performance.

## Discussion

DF is a common complication among patients with diabetes, particularly in those with a long duration of the disease, poor glycemic control, or multiple complications. As DF progresses to moderate or severe stages, the rate of amputations significantly increases. Overall, moderate to severe DFU and its related amputations represent a major global health concern, profoundly impacting patients’ quality of life. The factors contributing to moderate to severe DFUs are often multifactorial (14). While various risk factors have been identified, there remains controversy regarding their specific contributions. Furthermore, an accurate and clinically useful predictive model for assessing the risk of moderate to severe DFUs is currently lacking (15). To address this gap, the development of a predictive nomogram can provide intuitive, easily understandable, and practical data visualization tools. Such tools can assist healthcare providers in comprehending the risks associated with moderate to severe DFUs and making informed clinical decisions.

In our study, we conducted data analysis and identified nine independent risk factors, which we subsequently incorporated into a nomogram model to predict the risk of moderate to severe DFUs. The nomogram indicated that patients with DKD, DPN, DR, PAD, D-dimer, K-time, TC, LDL-C, and HDL-C were at an increased risk of moderate to severe DFUs among patient with T_2_DM. Using the area under the curve (AUC), calibration curve, Brier score, DCA and CIC, this model demonstrated superior discriminative ability, calibration, and clinical applicability.

Diabetic peripheral neuropathy (DPN) is a common complication of diabetes mellitus, affecting up to 50% of patients with long-standing diabetes. It is characterized by damage to the nerves, particularly the peripheral nerves, leading to symptoms such as pain, numbness, tingling, and loss of sensation in the extremities (16). The relationship between DPN and diabetic foot complications is significant. DPN can result in a loss of sensation in the feet, causing individuals to be unaware of injuries or pressure points that could lead to ulcers. The loss of protective pain sensation (LOPS) is a critical factor in the development of foot ulcers among people with diabetes. Additionally, the autonomic neuropathy often associated with DPN can lead to changes in sweat gland function and skin integrity, further predisposing individuals to ulceration (17). In a study conducted by S. Bajaj et al. (18) in northern India, it was found that 30% of patients with diabetes and new ulcers had neuro-ischemic ulcers (NIUs), whereas 70% had neuropathic ulcers. The prevalence of NIUs is on the rise, with other recent studies reporting rates ranging from 23.3% to 30.5% (19, 20). In Tanzania, it was observed that 100% of patients presenting with foot ulcers at a large Diabetes Outpatient Clinic exhibited varying degrees of peripheral neuropathy severity (21).

PAD is another significant comorbidity in patients with DFUs. Furthermore, The prevalence of PAD among patients with DFUs has also been a focus of investigation. A study by S. Vijayasarathy et al. (22) reported that 36% of patients with diabetic foot ulcers were found to have PAD. This was corroborated by the findings of Muthiah A et al., who demonstrated a prevalence of 38%, noting that out of 150 patients, 57 had PAD associated with diabetic foot ulceration (23). Our study also identified DPN and PAD as risk factors for the development of moderate to severe DFUs, consistent with prior research. For patients with diabetes, regular assessment of peripheral nerve function and foot health, along with evaluation for PAD, is essential for preventing the onset and progression of DFUs.

Our study revealed a significant association between DR and moderate to severe DFUs, which aligns with previous research. DFUs and DR are severe complications of Type 2 diabetes. While their pathogenesis has been debated, DR is generally considered to have a microvascular origin, whereas DFUs are more likely caused by a combination of microvascular and macrovascular disease. The significance of DR as a risk factor for developing DFUs has been documented by numerous investigators since 1975, when Walsh et al. (24) proposed a potential association between these complications. Some cohort studies have indicated that the risk of developing DFU is approximately doubled in participants with DR. As reviewed by Rossboth et al. (25), previous cohort studies have consistently identified a positive relationship between DR and subsequent DFUs. Borderie et al. (26) highlighted the persistence of this relationship even after adjusting for the effective International Working Group on the Diabetic Foot (IWGDF) classification. Specifically, they observed a five-fold increased risk of DFUs in subjects graded 0 and a two-fold risk for higher grades. Regardless of the underlying mechanism, the higher risk of DFUs in individuals with DR offers valuable information for clinicians aiming to prevent moderate to severe DFUs and amputations. Therefore, systematic screening for DR is recommended for patients with type 2 diabetes (27).

DKD is a major complication of diabetes mellitus, and it has a significant relationship with DF. The presence of DKD can exacerbate the risk and worsen the prognosis of DFUs. DKD can contribute to the development of DFUs through various pathophysiological mechanisms. These include the promotion of a pro-inflammatory state, the accumulation of advanced glycation end products, and the impairment of wound healing due to the uremic environment. Additionally, patients with DKD often have other comorbidities such as hypertension, which can further increase the risk of DFUs (28). In the context of our study, understanding the interplay between DKD and DFUs is crucial for identifying high-risk patients and implementing targeted interventions. Our findings indicate that patients with both DKD and additional risk factors have a significantly higher incidence of moderate to severe DFUs, underscoring the need for comprehensive assessments that include renal function and related complications. By incorporating DKD into our predictive model, we can enhance its accuracy and clinical applicability, ultimately aiding in early intervention strategies to prevent moderate to severe DFUs in at-risk populations.

The most novel findings of our study are the independent contributions of the coagulation markers, K-time and D-dimer, to the prediction of DFU severity. The relationship between D-dimer, K-time, and DFUs is complex and multifactorial. Elevated D-dimer levels, a marker of fibrin degradation and hypercoagulability, are associated with conditions common in DFUs, such as underlying inflammation, tissue damage, and an increased risk of thrombotic complications, all of which can exacerbate disease severity (29).

A shortened K-time signifies accelerated clot formation, a key feature of this prothrombotic phenotype (11). We hypothesize that this hypercoagulability contributes directly to DFU pathogenesis by promoting microvascular thrombosis. In the context of diabetic peripheral artery disease and endothelial dysfunction, an increased propensity for clot formation can lead to the occlusion of small vessels in the foot. This exacerbates critical limb ischemia, impairs microcirculatory perfusion, and compromises the delivery of oxygen and nutrients necessary for wound healing, thereby increasing the risk of ulcer development and progression to more severe disease stages. Thus, K-time serves as a functional measure of the hemostatic derangement underlying microvascular compromise in the diabetic foot (11). This pathophysiological mechanism provides a compelling biological rationale for why these markers are predictors in our model. In our study, we integrate both D-dimer and K-time into a predictive model to evaluate their collective role as risk factors for DFU development and progression. By analyzing their relationship with ulcer severity and outcomes, we aim to identify critical indicators for effective risk prediction. Further research is warranted to fully elucidate the clinical significance of these markers in DFU management.

Nehring et al (30). proved that hyperlipidemia (OR 0.54, 95% CI 0.36–0.81, P = 0.01) is the risk factor for the occurrence and development of diabetic foot. Dyslipidemia, characterized by low levels of HDL-C and elevated levels of LDL-C, has been identified as an independent risk factor for the development of DFUs. Low HDL-C levels can impair the body’s ability to transport cholesterol away from tissues, potentially leading to vascular complications, while high LDL-C levels contribute to atherosclerosis and reduced blood flow. These lipid abnormalities can exacerbate conditions such as neuropathy and peripheral artery disease, significantly increasing the risk of ulcer formation in diabetic patients.

Our study revealed counterintuitive associations between conventional lipid parameters and DFU risk, which warrant careful interpretation. The finding that higher levels of LDL-C appeared protective is likely not causal but may instead reflect a phenomenon known as the ‘lipid paradox’ or reverse epidemiology. Severe DFUs are characterized by a profound systemic inflammatory and catabolic state, which can suppress hepatic lipoprotein synthesis and lower circulating lipid concentrations. Thus, lower LDL-C levels may be a consequence of severe illness rather than a cause of protection (31–33). While intriguing, this association warrants cautious interpretation. It should not be construed as evidence against lipid management in diabetics. Instead, it highlights the complex interplay between lipid metabolism, systemic inflammation, and critical illness outcomes. Future prospective studies with detailed medication data and serial lipid measurements are needed to dissect these relationships.

This study had several limitations. First, its single-center, retrospective design inherently introduces the potential for unmeasured confounding and selection bias. Second, although internal validation showed outstanding performance, the exceptionally high AUC necessitates cautious interpretation and mandates external validation in a prospective, multi-center cohort to confirm generalizability. Third, we encountered initial statistical challenges with extreme odds ratios for some variables, which indicated potential separation; while we addressed this using Firth’s penalized-likelihood regression to obtain stable estimates, must be confirmed and explored in future studies. Finally, the sample size, though adequate for initial model development, is relatively small for a model with nine predictors, which may affect the precision and stability of the estimates. Future studies with larger sample sizes are needed to refine the model.

## Conclusion

In conclusion, our study presented a comprehensive predictive model for moderate to severe DFUs, incorporating nine clinically relevant risk factors. This model offers a valuable tool for healthcare providers to assess the risk of DFUs and make informed clinical decisions. Future research should focus on validating this model in diverse populations and exploring additional biomarkers that may further enhance its predictive accuracy. By identifying patients at high risk, we can target interventions more effectively, ultimately aiming to reduce the incidence of amputations and improve the quality of life for patients with diabetes.

## Data Availability

The datasets used or analyzed during the current study are available from the corresponding author on reasonable request. Requests to access these datasets should be directed to linjiayu@fjmu.edu.cn.
